# Integrated Surveys of Neglected Tropical Diseases in Southern Sudan: How Much Do They Cost and Can They Be Refined?

**DOI:** 10.1371/journal.pntd.0000745

**Published:** 2010-07-13

**Authors:** Jan H. Kolaczinski, Kara Hanson, Emily Robinson, Diana Picon, Anthony Sabasio, Martin Mpakateni, Mounir Lado, Stephen Moore, Nora Petty, Simon Brooker

**Affiliations:** 1 Malaria Consortium – Africa Regional Office, Kampala, Uganda; 2 London School of Hygiene and Tropical Medicine, London, United Kingdom; 3 Malaria Consortium, Juba, Southern Sudan; 4 Ministry of Health, Government of Southern Sudan, Juba, Southern Sudan; 5 Kenya Medical Research Institute (KEMRI)-Wellcome Trust Research Programme, Nairobi, Kenya; Ministry of Health, Uganda

## Abstract

**Background:**

Increasing emphasis on integrated control of neglected tropical diseases (NTDs) requires identification of co-endemic areas. Integrated surveys for lymphatic filariasis (LF), schistosomiasis and soil-transmitted helminth (STH) infection have been recommended for this purpose. Integrated survey designs inevitably involve balancing the costs of surveys against accuracy of classifying areas for treatment, so-called implementation units (IUs). This requires an understanding of the main cost drivers and of how operating procedures may affect both cost and accuracy of surveys. Here we report a detailed cost analysis of the first round of integrated NTD surveys in Southern Sudan.

**Methods and Findings:**

Financial and economic costs were estimated from financial expenditure records and interviews with survey staff using an ingredients approach. The main outcome was cost per IU surveyed. Uncertain variables were subjected to univariate sensitivity analysis and the effects of modifying standard operating procedures were explored. The average economic cost per IU surveyed was USD 40,206 or USD 9,573, depending on the size of the IU. The major cost drivers were two key categories of recurrent costs: i) survey consumables, and ii) personnel.

**Conclusion:**

The cost of integrated surveys in Southern Sudan could be reduced by surveying larger administrative areas for LF. If this approach was taken, the estimated economic cost of completing LF, schistosomiasis and STH mapping in Southern Sudan would amount to USD 1.6 million. The methodological detail and costing template provided here could be used to generate cost estimates in other settings and readily compare these to the present study, and may help budget for integrated and single NTDs surveys elsewhere.

## Author Summary

Control of neglected tropical diseases (NTDs) is suggested to be more cost-effective when drugs are co-administered through a single integrated delivery system rather than separate systems. An essential prerequisite for such efficiency gains is sufficient geographical overlap of the targeted diseases – lymphatic filariasis (LF), onchocerciasis, schistosomiasis, soil-transmitted helminth infection and trachoma. Lack of data on geographical NTD distribution currently hampers the implementation of integrated control in many African countries. To generate the required data quickly and efficiently, integrated surveys of several NTDs simultaneously have been recommended. However, experience with integrated surveys is limited and requires additional research on cost and effectiveness to inform improvements in methodology and to guide scale-up. Here we analyse costs of the first integrated NTD survey round in Southern Sudan, generating average costs per implementation unit surveyed. Cost estimates are presented for use of the existing survey method and for modified versions. Key cost drivers were survey consumables and personnel, both of which are recurrent costs. These inputs could be reduced or put to more efficient use by modifying sampling for LF. To generate comparable cost estimates and identify key cost drivers in other settings we provide detailed cost data and guidance on how to replicate this work.

## Introduction

Health intervention needs generally exceed available funds. Managers of disease control programmes therefore need to decide how to allocate their resources most efficiently. This is particularly so for the control and elimination of neglected tropical diseases (NTDs), which have been chronically underfunded [1], [2]. In an attempt to increase the efficiency of NTD programmes, the co-administration of preventive chemotherapy (PCT) for lymphatic filariasis (LF), onchocerciasis, schistosomiasis, soil-transmitted helminth (STH) infections and trachoma is widely advocated, the rationale being that in sub-Saharan Africa (SSA) the distributions of these diseases often overlap [3]. In areas of NTD co-endemicity one delivery structure, instead of several, could therefore be used for mass drug administration (MDA) of PCT [4], [5].

Co-endemicity, the prerequisite for the anticipated efficiency gains of integrated control, applies not at the country level, but at sub-national levels where climatic and other determinants are suitable for the transmission of more than one of above diseases [6], [7]. For example, onchocerciasis is associated with fast flowing rivers [8], schistosomiasis occurs near calmer or stagnant waterbodies [9], whilst STH infection, trachoma and LF occur over relatively large areas [10]–[12]. Because of these differing transmission ecologies, prevalence data on each disease are required to identify areas of overlap and to target these with integrated PCT delivery [13].

For large areas of SSA there are either no data to assess the potential for integration, or they are incomplete or out of date. Only the distribution of onchocerciasis has been comprehensively mapped, while LF mapping is ongoing in several countries and has not commenced in Chad and Eritrea [14]. In 2000, prevalence data for schistosomiasis and STH infections were only available for a third of all districts in SSA [15] and, in spite of increased resources for mapping, survey coverage remains patchy or absent in many areas [16]. No trachoma data were available for seven countries in the Africa region in 2005, while only a few countries had undertaken national surveys [17]. Although more NTD data are now being collected, the gaps in the known distributions are still considerable.

Given that funds are not just limited for intervention, but are particularly hard to mobilize for apparent ‘research’, some control programmes have started to survey multiple NTDs simultaneously instead of mapping the different diseases separately [18], [19]. The underlying rationale for such integrated surveys is the same as that for co-administration of PCT; reaching communities in SSA is often challenging and associated with considerable costs, so avoiding repeated access to conduct similar activities is likely to minimize the investment required to achieve a desired outcome, be it classification of an administrative area (e.g. district) for intervention or curing people from NTD infection. As yet there is limited operational experience with integrated NTD surveys, but it is clear that their design needs to balance cost against the precision and accuracy with which administrative areas are classified according to treatment needs.

To improve on current designs it is important to understand the main drivers of survey costs and investigate potential effects of modifying standard operating procedures. One such modification is altering the number of sites sampled, which is likely to affect both the cost and accuracy of determining whether a geographical area needs to be targeted with interventions. Such cost analyses should be undertaken using an approach that is ‘generalisable’, hence allowing comparison between settings and use of results to plan and budget for similar undertakings elsewhere [20], [21].

Southern Sudan, along with the Democratic Republic of Congo and Central African Republic, possibly has the largest unmapped NTD burden in SSA and hence the greatest need for up-to-date data. In 2008, based on available information [6], [22], Southern Sudan developed a national strategy for the integrated control of onchocerciasis, LF, schistosomiasis, STH infection and trachoma. An essential component of this strategy is to generate data on the distribution and co-distribution of the targeted NTDs (LF, schistosomiasis, STH infection nationwide, and trachoma in remaining regions). In 2009, an integrated NTD survey was conducted in Northern Bahr-el-Ghazal State, the first of ten states in the country [19], with completion of mapping in the remaining nine states planned for 2010/11.

The aims of this paper are to analyse the costs of the 2009 survey, to identify the main cost drivers, and to estimate the resources required to expand surveys to the whole of Southern Sudan. In addition, to help compare our results to those from other settings and estimate the costs of integrated surveys elsewhere, we present a standardised approach to costing integrated NTD surveys.

## Methods

### Integrated NTD Control in Southern Sudan

During 2007, the Ministry of Health, Government of Southern Sudan (MoH-GoSS), conducted a situation analysis of NTDs and their control in order to inform planning for NTD control and elimination. This analysis indicated that 12 NTDs were endemic, including all of the diseases for which MDA of PCT forms an important component of control, namely onchocerciasis, LF, schistosomiasis, STH infection and trachoma [22]. At the time, only onchocerciasis and trachoma had benefitted from regular MDA in some endemic areas, while STH infections in children had been treated through a number of deworming rounds alongside national immunization days. Although the need to control all NTDs endemic to Southern Sudan's was highlighted, an opportunity was identified to combine those diseases suitable for MDA-based control under an umbrella National Integrated NTD Control Programme. The aim of this undertaking was to increase geographical PCT coverage and the number of diseases treated in each location by expanding the scope of existing community-based delivery mechanisms, be it for NTDs or other interventions.

Two delivery mechanisms were identified for initial integration, the volunteer networks for community-directed treatment with ivermectin (CDTI, covering parts of the onchocerciasis endemic areas) and similar networks for Guinea worm eradication (which in some areas also delivers trachoma interventions). Given the lack of experience of expanding these networks into delivery platforms for PCT packages, the approach was to be piloted and gradually scaled up building on implementation experience gained along the way. In areas where neither CDTI nor the Guinea worm volunteer network are present, other existing delivery structures may need to be supported to take on integrated NTD control. Alternatively a new platform may need to be established to deliver MDA amongst other public health interventions [22].

### Implementation Units for Integrated NTD Control

Southern Sudan has four administrative tiers: state (1^st^), county (2^nd^), payam (3^rd^) and boma (4^th^). A county is the administrative unit most comparable to a district in other African countries. The majority of counties include five or more payams. In 2005, Southern Sudan had 49 counties, the majority of which were <10,000 km^2^ in size (median of 8,033 km^2^) and had a population of 100,000 to 500,000 inhabitants. At the same time these counties were divided into a total of approximately 308 payams that varied greatly in population and size. The overall median population of payams was 31,607, but this ranged from 2,000 to 120,000 inhabitants. The median payam size was 1,876 km^2^, ranging from 126 km^2^ (Cueibet in Lakes State) to 58,210 km^2^ (Raja in Western Bahr-el-Ghazal State).

The district, or an area of equivalent size, is the recommended implementation unit (IU) for LF elimination [23]. An area this size, however, may be too large for co-administration of a standardized drug package, because schistosomiasis and onchocerciasis are unlikely to be endemic throughout. The MoH-GoSS therefore decided to consider both the payam and the county as IUs of the Integrated NTD Control Programme.

### Survey Description

A large-scale survey of LF, schistosomiasis, STH infection and loiasis was conducted in Northern Bahr-el-Ghazal State between February and May 2009. Details of the study area and survey protocol are provided elsewhere [19], [24]. In summary, quasi-random two-stage cluster sampling was used to select communities on the basis of potential risk of LF and schistosomiasis and to randomly select households within these communities. Each household head was requested to provide written consent, and all children aged 5 to 16 years were asked to give verbal consent before providing stool and urine samples for examination of schistosome and STH infection; adults were only sampled for LF testing using immunochromatographic tests (ICT, BinaxNOW Filariasis, Inverness Medical).

For LF, up to three communities and 250 individuals per payam were sampled. Sites selected for LF were also sampled for schistosomiasis and STH, as well as loiasis, a disease whose presence complicates LF elimination [25]. In each payam, between three to four additional communities were sampled for STH and schistosomiasis, with the actual number depending on payam size and estimated population. A total of 43 communities were sampled for LF infections and 73 communities for schistosomiasis and STH infections. Communities were selected from each of the five counties of Northern Bahr-e-Ghazal State, with only one out of 22 payams not surveyed. Due to delays in the supply of ICTs for LF, the survey had to be conducted in two phases, lasting 22 and 31 days respectively, with a gap of three weeks in-between. Because of this delay, a total of 13 sites had to be revisited for LF data collection. As this delay was not part of standard operating procedures, we excluded the cost associated with re-visiting these communities, though the test-specific costs were included.

The composition of survey teams varied depending on the number of NTDs to be surveyed in each location. Where all four NTDs were surveyed, teams included two drivers, one supervisor, one interviewer/translator, and up to four technicians, travelling in two vehicles. One or two technicians undertook blood sampling and LF testing while the other two to three prepared and read stool and urine samples. For those sites where schistosomiasis and STH infection or just LF were surveyed, the team consisted out of one driver, one supervisor, one interviewer/translator, and at least two laboratory technicians, travelling in one vehicle. Due to severe human resource constraints affecting Southern Sudan, only two national laboratory technicians per team could be recruited, with additional technicians recruited as short-term consultants from the Vector Control Division, MoH, Uganda. Village guides were recruited locally.

Owing to the poor infrastructure in Northern Bahr-el-Ghazal State and fluctuating security, teams established camps in locations that were centrally located between study communities. In many cases, space to pitch tents was provided by non-governmental or faith-based organizations in their compounds, but a charge was levied. Otherwise teams stayed in local guesthouses. For camping, sleeping and cooking equipment, food, fuel and small generators were procured. Three Toyota Land Cruisers were used during the surveys.

To prepare for the surveys, ten days were needed to arrange supplies and develop the database, a total of four days were required to move vehicles from Juba to Northern Bahr-el-Ghazal State and back, and two days were required to train surveyors on the study protocol. At the end of the survey, one day was allocated to take stock and clean and store supplies and equipment. After completion of all survey activities, five days were needed to clean the data and undertake preliminary analysis.

### Collection of Cost Data

Both financial and economic costs were estimated from the perspective of the provider [26], in this case Malaria Consortium and the MoH-GoSS. Financial costs were the cash expenditures made to enable implementation of the survey. For capital items, these were estimated for the total number of survey days by means of straight-line depreciation followed by calculation of an average financial daily cost. Economic costs captured the value of all resources consumed by the survey, including opportunity costs of volunteers and equipment that were used in the survey but not paid for, as well as appropriate treatment of costs of capital items with a value of >USD 100 and an expected useful life of more than one year [27]. Costs of MoH staff were based on the GoSS pay scale for 2009 while the time of international volunteers was valued using equivalent Malaria Consortium salaries for this setting. Capital items were discounted over their estimated useful life using the recommended discount rate of 3% [28], [29]. Daily economic costs were calculated for all capital items and multiplied by the appropriate number of days in use during the survey. Based on our experience of working in the harsh climatic environment of Southern Sudan we estimated the useful life to be four years for vehicles and high frequency radios (fitted to vehicles) and two years for all other items, including laptop computers and laboratory field equipment. All resources used for research activities, such as this costing study, were excluded from the analysis as were the costs associated with the development of the survey protocol.

Cost data were collated from financial expenditure records of Malaria Consortium during the survey and shortly afterwards. To accommodate considerable fluctuations in currency conversion rates, two time periods were used. For payments for survey supplies, which started in October 2008 and continued through May 2009, we used average exchange rates of 1 United States Dollars (USD) = 0.67 British Pounds (GBP) or 1 USD = 2024 Ugandan Shillings (UGX). For costs associated with the actual survey activities between February and May 2009 we used a rate of 1 USD = 2.3 Sudanese Pounds (SDG) (http://www.oanda.com/convert/fxhistory). We assumed that these exchange rates and the wages paid reflected competitive foreign exchange markets, and therefore did not use shadow prices to adjust for possible distortions [30].

Costs were identified using an ingredients approach, whereby the total value of each of the services and goods employed in implementing the survey was estimated by identifying the number of units consumed and multiplying these by their unit price [26], [31]. Following the structure of the survey, cost and services were organized into capital and recurrent costs, both of which were divided into cost categories [Box 1, Dataset S1].

It is common practice to include overheads in cost analyses [32]. To capture the indirect costs of project management and administration we applied an overhead of 25% to the financial cost estimate for all budget lines. This rate is higher than those applicable to operations in more stable settings of eastern Africa [e.g. [33], but in our experience provides an accurate reflection of the cost involved in implementing programmes in a land-locked country undergoing post-conflict reconstruction.

### Outcomes

The overall purpose of the integrated survey was to generate data by which IUs could be classified according to intervention thresholds recommended by the WHO [3]. The present analysis considered both the county and the payam as IUs, because a two-tier system may be needed to account for inherent differences in the geographical distribution of the targeted NTDs. While for LF elimination the county is likely to be an appropriate IU, control of onchocerciasis is already more geographically focused and the first round of integrated NTD surveys indicated that a similar approach is also more appropriate for schistosomiasis [19]. The outcomes used for our costing study were thus the county and the payam surveyed, to allow their classification for intervention according to WHO recommended thresholds.

### Sensitivity Analysis

Calculation of costs and outcomes involves a number of assumptions; the ones underlying the present study have been outlined above and in table 1. One-way sensitivity analysis was conducted to explore the effects of key assumptions on the results. We varied the discount rate (reduced to 0% or increased to 10%) and increased the assumed lifespan of vehicles to 7.5 years. In addition we investigated the effect of modifying the standard operating procedures. Instead of using Hemastix reagent strips validated by urine filtration for diagnosis of urinary schistosomiasis, as used in the first survey round [34], we assumed that urinary schistosomiasis could be adequately diagnosed using reagent strips only and excluded the cost of urine filtration, a method that is relatively costly because it requires isopore membrane filters at a price of nearly USD 2 per filter. The rationale for cutting out urine filtration was that the use of reagent strips alone is a reliable diagnostic procedure in some African countries [35] and may, after validation, be applicable in high schistosomiasis transmission settings in Southern Sudan. We also explored the effect on cost of classifying counties, rather than payams, for LF elimination. Instead of sampling 250 individuals from up to three sites per payam, the analysis investigated the cost implications of applying exactly the same procedure but at county level. This procedure would have yielded the same classification of IUs for LF elimination in Northern Bahr-el-Ghazal State [19] and is likely to be appropriate throughout Southern Sudan.

**Table 1:** Reference case scenario.

Parameter	Suggested Reference Scenario	Explanation
Perspective	Provider	Use provider perspective unless considerable survey contributions were made by other parties and resources are available to measure all of these inputs, including the opportunity cost of survey participants. In this case present results from both the provider and societal viewpoints.
Output	Implementation unit surveyed	Implementation unit (e.g. district) surveyed to allow classification for intervention according to WHO recommended thresholds [3]. Provide indication of the geographic and population size of the implementation unit.
Cost data	Include:All survey costs (e.g. wages, transport)Time cost of survey staff, including volunteersTransportation and other non-medical servicesDonated itemsOverhead costs	Use ingredient approach with cost categories as suggested in box 1.Quantities and prices need to be presented separatelyExclude research costs.Include relevant overheads of collaborating organizations (e.g. NGO contributions to procurement, management, etc), if relevant.Treat any equipment as a capital item if it is expected to last for more than one year and is purchased at a value of USD 100 or more.Calculate a daily financial cost for capital items by using straight line depreciation, and include the cost of each capital item for the number of survey days that it was used.
Currency	US$	Costs should be presented in US$, indicating the year of conversion.
Lifespan of capital items	Vehicles: 4 yearsOther equipment: 2 years	Establish average lifespan of capital items under local conditions and provide details on these in the narrative. Explore the impact of these assumptions in the sensitivity analysis.
Adjustment of financial costs to calculated economic costs		
Annualisation	Lifespan of capital items as specified above	To obtain an equivalent annual cost for each capital outlay, an annualisation procedure needs to be followed. This requires an estimate of the lifespan of each capital item and a decision on the discount rate to be used (see below).A proportion of the annualised costs should be included as economic costs. We recommend that time (i.e. days of use of the equipment) is used for this calculation.
Discount rate	3%	Base-case calculations should use 3%, to be consistent with World Bank recommendations [32]. This should be varied in the sensitivity analysis, e.g. from 0 – 10%.
Reporting of results		
Cost estimate	Cost per implementation unit surveyed	Provide costs in US$Specify year in which costs were calculated or adjusted to

### Ethical Considerations

The present study involved collection of data on cost and non-financial inputs through analysis of Malaria Consortium expenditures and activities during an epidemiological survey. Data collection was conducted from the ‘provider perspective’, rather than the all encompassing ‘societal perspective’ [26], since participation in the survey incurred minimal time commitment from the study communities. The epidemiological survey itself received ethical approval from the Directorate of Research, Planning and Health System Development, MoH-GoSS, and from the Ethics Committee of the London School of Hygiene and Tropical Medicine, UK. Collection of the data presented here did not involve human subjects and therefore did not require ethical approval.

## Results

The total financial costs for the survey amounted to USD 182,067, the majority of which was spent on recurrent items. The principal cost drivers were personnel (34.4%) followed by survey consumables (30.4%) (Table 2). Economic costs amounted to a total of USD 201,030 and were arrived at by including the imputed value of the time provided by MoH employees and other non-cash inputs, as well as the opportunity cost of capital items used in the survey. Because all assets above USD 100 in value and with a lifespan >1 year were annualised and only their time in use during the survey was included, capital cost amounted to only 4.3% of the total survey costs and were largely comprised of vehicle costs. As for the financial costs, most economic costs were taken up by survey consumables and personnel, accounting for 27% and 38% of the total, respectively (Table 2).

**Table 2:** Financial and economic costs, and cost profiles, for an integrated NTD survey in Northern Bahr-el-Ghazal State.

	Total financial cost (USD)	% of financial cost*	Total economic cost (USD)	% of economic cost*
**Capital costs**				
Vehicles	4,187	2.9%	4,504	2.7%
Communication & IT equipment	858	0.6%	906	0.6%
Accommodation equipment	378	0.3%	395	0.2%
Survey equipment	776	0.5%	1,206	0.7%
**Subtotal**	**6,199**	**4.3%**	**7,011**	**4.3%**
**Recurrent costs**				
Travel	15,183	10.4%	18,688	11.4%
Vehicle fuel & maintenance	10,635	7.3%	10,635	6.5%
Accommodation & sustenance	18,188	12.5%	20,688	12.6%
Survey consumables	44,346	30.4%	44,346	26.9%
Communication	1,019	0.7%	1,019	0.6%
Personnel	50,084	34.4%	62,230	37.8%
**Subtotal**	**139,455**	**95.7%**	**157,606**	**95.7%**
**Subtotal**	**145,653**		**164,617**	
*Overhead (25%)*	*36,413*		*36,413*	
**Total**	**182,067**		**201,030**	
Counties surveyed			5	
Payams surveyed			21	
**Cost per county classified for implementation**			**40,206**	
**Cost per payam classified for implementation**			**9,573**	

*: Proportions were calculated on the subtotal, i.e. excluding the overhead.

The outcome from this investment was that 21 payams in the five counties of Northern Bahr-el-Ghazal State, an area the size of Belgium, were surveyed for LF, schistosomiasis, STH infection and loiasis. These five counties and 21 payams were, for the first time ever, categorised as requiring MDA delivery or not, according to WHO recommended thresholds [3]. The average economic cost per IU classified was USD 40,206 per county or USD 9,573 per payam. These estimates changed very little when the two key assumptions, the discount rate or lifespan of vehicles, were varied (Table 3).

**Table 3:** Sensitivity of cost estimates to underlying assumptions or modification of routine operating procedures.

Assumptions tested	USD/county classified	% deviation from base case	USD/payam classified	% deviation from base case
Base case	40,206	-	9,573	-
Discount rate:				
0%	40,119	−0.2%	9,552	−0.2%
10%	40,417	+0.5%	9,623	+0.5%
Vehicle lifespan:				
7.5 years	39,721	−1.2%	9,457	−1.2%
Urinary schistosomiasis diagnosis:				
Dipstick test only	39,379	−2.1%	9,376	−2.1%
Testing for LF infection:				
County level only	30,291	−24.7%	7,212	−24.7%

Survey costs could be lowered if the standard operating procedure were modified. Testing for urinary schistosomiasis infection could, for example, be conducted only with Hemastix reagent sticks (Bayer Diagnostics, Basingstoke, UK), instead of the current procedure whereby all samples with blood in urine and a sub-sample of negatives were cross checked using urine filtration. By cutting out the cost of these and other supplies for urine filtration, the cost per implementation unit surveyed would have decreased by 2% (Table 3). This small reduction in economic cost is explained by the fact that urine filtration can be easily conducted alongside other operations; personnel requirements (the major cost driver) and survey duration would thus remain unchanged.

In Northern Bahr-el-Ghazal State, where LF was found not to be endemic, sampling of up to 250 individuals from up to three sites per county would have resulted in the same classification of IUs. We therefore proposed that testing for LF infection is from now on conducted at the county instead of payam level throughout Southern Sudan, given that LF tends to be endemic over relatively large areas [12]. Sensitivity and specificity of LF detection, and hence correctly classifying IUs for interventions, is unlikely to be affected by surveying areas larger than the payam. This approach would also be more consistent with WHO recommendations for establishment and sampling of IUs for LF [23], as size and administrative level of the county in Southern Sudan is equivalent to that of the district in other African countries. If the standard operating procedures of the survey were thus modified, quantities of ICT tests, safety lancets and other supplies required to safely take blood samples would be reduced by approximately 80%, incurring substantial savings on survey consumables, particularly ICTs that cost >USD 4.0 per test. Furthermore, existing vehicles and staff could be deployed in three teams, which would decrease the survey duration by about one day per payam and reduced the majority of running costs accordingly. The cost per IU classified could thus be reduced by about 25% without any apparent risk of missing LF foci.

Based on the data presented here we estimate that completion of NTD mapping in Southern Sudan by using an integrated NTD survey design would amount to an economic cost of approximately USD 1.6 million. This calculation is based on the assumption that LF surveys would only be conducted at county level and that the administrative divisions used to conduct National Immunization Days (NIDs) in 2008 would be used to identify survey areas. At the time of the 2008 NIDs there were a total of 58 counties, of which 53 remain to be surveyed for NTDs today.

## Discussion

The present work was primarily conducted to generate a better understanding of the costs of conducting integrated NTD surveys in areas of Southern Sudan where there are no or little data on LF, schistosomiasis, STH infection and loiasis. In addition, we aimed at providing sufficient methodological insight and guidance to allow other researchers and programme managers to repeat similar costing exercises or to estimate the financial cost of doing similar surveys in their own setting.

We found the major cost drivers to be two categories of recurrent costs – laboratory consumables and personnel. Currently in Southern Sudan, both of these inputs largely have to be imported by the project, due to the lack of a viable commercial sector and severe human resource constraints. The cost of survey consumables is likely to be similar in other African countries, as most of them are not produced in the region, while personnel costs might differ considerably between settings, particularly when comparing Southern Sudan to countries that have not been affected by decades of conflict. In Southern Sudan, the need for considerable technical support meant that personnel accounted for 34% of the total survey cost. Interestingly, when we simulate survey costs for Uganda, by supplementing our daily rates paid to laboratory technicians with the official perdiem plus salary rates applied by the MoH in Uganda and by excluding the cost of international flights associated with bringing laboratory technicians into Southern Sudan, we found that survey costs were very similar. This somewhat surprising finding indicates that more data on the particular features of different settings and more detailed analyses are clearly needed to clarify whether NTD surveys in Southern Sudan are no more expensive than in other countries. Such studies will also confirm whether survey supplies and personnel are generally the key cost drivers. For now we suggest that these two inputs should be budgeted in considerable detail when NTD surveys are planned elsewhere, as our results indicate that they are likely to account for the greatest share of costs.

To generate further evidence on survey costs and the ingredients that drive these, we have provided considerable detail on the present analysis (Box 1, Dataset S1) and would like to propose a reference case scenario (Table 1). Application of this template by other researchers and programme managers would assure that results from future costing studies could be readily compared. Depending on the specific modes of survey implementation additional analyses, for example use of the societal perspective, may be more appropriate in these settings; different assumptions, such as a longer lifespan of capital items, might be more appropriate. While we encourage such analysis, we suggest that these results are reported in addition to those obtained using the proposed reference case scenario (Table 1), to allow direct comparison of costing results between studies.

In Southern Sudan, nine more states need to be surveyed for LF, schistosomiasis and STH infection before all endemic areas can be targeted with appropriate intervention packages. The current relative political stability provides a good opportunity to do so, and survey activities should thus be scaled up to finish this job as quickly as possible. We found that this could be done at slightly lower cost per IU classified, if the standard operating procedures used in Northern Bahr-el-Ghazal State were somewhat modified while continuing to conform with WHO recommendations [23], [36]. For diagnosis of urinary schistosomiasis, moderate cost saving could be obtained by limiting methods to the use of reagent strips for detection of blood in urine, a proxy for infection with *Schistosoma haematobium* [37]. In Southern Sudan, however, this modification would result in considerable misclassification of IUs for treatment with praziquantel owing to the relatively low specificity of Hemastix reagent strips in this setting [34]. A more meaningful modification of the standard operating procedures would be to reduce the number of individuals and sites sampled for LF. For this disease the county, rather than the payam, seems the appropriate IU. It is unlikely that sampling LF at the county level would fail to identify endemic foci, because in most settings the disease tends to occur over relatively large geographical areas [12] and survey sites are purposefully selected using information on the occurrence of lymphoedema and hydrocoele [23]. Apart from the immediate benefit of reducing the cost of survey consumables and speeding up operations, LF sampling at the county level would have the added advantage of reducing thermal storage requirements for ICT kits.

The present costing study of an integrated NTD survey is the first of its kind, which somewhat limits our ability to generalize the findings even within the same context. Starting up new activities tends to require more preparation, training and supervision than routine operations. This first round of surveys could thus be considered a ‘start-up period’ for NTD mapping in Southern Sudan, where important practical experience was gained. Ideal team composition, allocation of task and establishment of smooth workflows took some time to establish. Subsequent survey rounds are thus likely to run smoother and faster, unless they were to be conducted during the rainy season. Our cost estimates are therefore likely to be conservative, although the analysis accounted for one of the major delays experienced – the need to resample a selection of sites for LF. Nevertheless, based on our experience of operating in the challenging terrain of Southern Sudan, we would still recommend conservative budgeting, because field activities are always subject to considerable uncertainty. Weather conditions frequently delay access and supplies, and demands for higher salaries and incentives are rapidly increasing during present post-conflict reconstruction. Future costing work will tell whether economies of scale [38] apply to integrated NTD surveys in this setting.

### Conclusion

The major cost drivers of an integrated NTD survey in Southern Sudan were personnel and consumables. It is likely that this observation is applicable to most other settings, and should be taken into account during the budgeting process in countries wishing to apply this survey approach. Overall costs may be lower in settings that have not recently experienced conflict, because there will be less need to draw on international staff for technical support and overall operating costs may be lower. To confirm or refute this assumption we encourage further costing work on integrated, as well as single NTD surveys, using the approach outlined here. Most importantly, such analysis should help to identify areas of potential cost savings and be able to provide an estimate of the overall investment required to complete NTD mapping for specific areas.[Table pntd-0000745-t004]

**Box 1. Cost Categories Used and Ingredients Included**

**Table pntd-0000745-t004:** 

Type/Category	Ingredients
**Capital costs**	
Vehicles	Vehicles, including freight charges Vehicle parts with a unit cost of USD 100 or more Any vehicle equipment expected to last for more than one year (e.g. first aid kits)
Communications & IT Equipment	High Frequency (HF) and Very High Frequency (VHF) radios Phones (fixed, mobile, satellite) Global Positioning System (GPS) devices, Geographic Information Systems (GIS) software and manuals Computers
Accommodation equipment	Camping equipment estimated to last >1 year Generator
Survey equipment	Microscopes Laboratory glassware and storage containers Furniture (i.e. plastic tables and chairs) All durable equipment for Kato-Katz test and urine filtration
**Recurrent costs**	
Travel	Airfares & short-term vehicle hire (e.g. during preparatory activities) Per-diems Visas and other in-country registration fees Accommodation during travel to survey area Travel insurance and pre-travel medical expenses Fuel costs associated with moving vehicles to survey area
Fuel/Maintenance	Fuel for vehicles during field work & all costs associated with obtaining the fuel, such as loading/unloading of fuel barrels Any maintenance costs, including parts with a unit cost below USD 100 Vehicle insurance
Accommodation & sustenance	Rental of guesthouse Hotel bills All costs associated with purchasing and preparing food Cook and guesthouse guards Camping equipment estimated to last <1 year
Survey consumables	Laboratory consumables, including rapid diagnostic tests and drugs to treat survey participants Freight charges for import of consumables Stationary items T-shirts and other types of identification worn by members of survey team
Communication	Mobile and satellite phone credit
Personnel	Salaries paid to drivers, supervisors, laboratory staff, village guides and consultants Field allowances Staff time for cross-checking of slides Technical support time for data entry and analysis

## Supporting Information

Dataset S1 Details on inputs, quantities, and associated costs that were required for implementation of integrated NTD surveys in Northern Bahr-el-Ghazal State, Southern Sudan.
(0.23 MB DOC)
