# Supplementary material for: Integrated Surveys of Neglected Tropical Diseases in Southern Sudan: How Much Do They Cost and Can They Be Refined?
Source: PLoS Negl Trop Dis. 2010 Jul 13;4(7):e745. doi: 10.1371/journal.pntd.0000745 (PMC2903472; doi:10.1371/journal.pntd.0000745)
Supplement: Dataset S1 — Details on inputs, quantities, and associated costs that were required for implementation of integrated NTD surveys in Northern Bahr-el-Ghazal State, Southern Sudan. (0.23 MB DOC) [file pntd.0000745.s001.doc]

Dataset S1 Details on inputs, quantities, and associated costs that were required for implementation of integrated NTD surveys in Northern Bahr-el-Ghazal State, Southern Sudan

Part A: CAPITAL COSTS

| INPUT | UNIT | QUANTITY | DAILY UNIT COST (USD) | COST TYPE[[1]](#footnote-2) | TOTAL DAYS OF USE[[2]](#footnote-3) |
| --- | --- | --- | --- | --- | --- |
| Vehicles |  |  |  |  |  |
| Toyota Land Cruiser | Vehicle (incl. shipping) | 3 | 30.4 (28.2) | E (F) | 146 |
| Vehicle first aid kits | Kit | 3 | 0.15 (0.14) | E (F) | 146 |
| Shock absorber | Vehicle part | 2 | 0.43 (0.41) | E (F) | 114 |
| *Sub-total* | *USD 4,504 (4,187)**[[3]](#footnote-4)* | | | *E (F)* |  |
| Communications & IT equipment |  |  |  |  |  |
| HF communication equipment for vehicles | Comms per car (including labour) | 3 | 2.3 (2.1) | E (F) | 146 |
| Thuraya Satelite phone | Phone | 3 | 1.2 (1.2) | E (F) | 180 |
| Mobile Phones | Phone | 3 | 0.05 (0.04) | E (F) | 180 |
| GPS | Handheld GPS | 3 | 0.15 (0.14) | E (F) | 180 |
| GIS software (ArcView) | Software CD | 2 | 1.9 (1.8) | E (F) | 10 |
| Arcview manual | Manual | 1 | 0.11 (0.10) | E (F) | 5 |
| Laptop computer | Computer | 2 | 1.7 (1.6) | E (F) | 174 |
| *Sub-total* |  | *USD 906 (858)Error: Reference source not found* | | *E (F)* |  |
| Accommodation equipment | |  |  |  |  |
| Storage boxes | Box | 20 | 0.09 (0.09) | E (F) | 1200 |
| Small generators | Generator | 2 | 1.8 (1.7) | E (F) | 112 |
| Plastic wash basins | Basin | 20 | 0.01 (0.01) | E (F) | 1120 |
| Cooking Utensils | Utensil set | 5 | 0.15 (0.14) | E (F) | 280 |
| Dinner plates | Set | 1 | 0.08 (0.07) | E (F) | 56 |
| Frying pan | Pan | 1 | 0.02 (0.02) | E (F) | 56 |
| Charcoal stove | Stove | 3 | 0.05 (0.04) | E (F) | 168 |
| Jerry cans | Can | 19 | 0.01 (0.01) | E (F) | 1064 |
| Water bottles | Bottle | 15 | 0.01 (0.01) | E (F) | 840 |
| Torches | Torch | 16 | 0.01 (0.01) | E (F) | 896 |
| *Sub-total* |  | *USD 395 (378)Error: Reference source not found* | | *E (F)* |  |
| Survey equipment |  |  |  |  |  |
| Clipboards | Clipboard | 10 | 0.01 (0.01) | E (F) | 560 |
| Rucksacks for team | Rucksack | 15 | 0.02 (0.02) | E (F) | 840 |
| Foldable tables for lab | Table | 8 | 0.03 (0.03) | E (F) | 448 |
| Foldable stools for lab | Stool | 12 | 0.02 (0.02) | E (F) | 672 |
| Buckets | Bucket | 10 | 0.01 (0.01) | E (F) | 560 |
| Scissors | Scissors | 2 | 0.01 (0.01) | E (F) | 112 |
| Storage bottle 250ml | Flask | 2 | 0.1 (0.09) | E (F) | 112 |
| Tweezers | Pair | 8 | 0.02 (0.02) | E (F) | 448 |
| Microscope set (Olympus CX 21 EIM), mirror & storage box | Microscope | 3 | 2.25 (2.16) | E (F) | 168 |

| INPUT | UNIT | QUANTITY | DAILY UNIT COST (USD) | COST TYPEError: Reference source not found | TOTAL DAYS OF USEError: Reference source not found |
| --- | --- | --- | --- | --- | --- |
| CAPITAL COSTS: |  |  |  |  |  |
| Survey equipment (continued) | |  |  |  |  |
| Microscopes on loan from WHO | Microscope | 3 | 2.25 | E | 168 |
| Dose poles | Pole | 3 | 0.04 (0.04) | E (F) | 168 |
| Tally counter | Counter | 12 | 0.04 (0.03) | E (F) | 672 |
| Slide storage boxes | Box | 12 | 0.02 (0.02) | E (F) | 672 |
| Kato katz templates (borrowed from VCD) | Kit | 1 | 0.29 | E | 56 |
| Measuring cylinder | Cylinder 50ml | 6 | 0.01 (0.01) | E (F) | 336 |
| Measuring cylinder | Cylinder 100ml | 6 | 0.01 (0.01) | E (F) | 336 |
| Woven wire stainless steel mesh sieve | Sieve | 12 | 0.13 (0.12) | E (F) | 672 |
| Swinnex filter holder 13mm without filter | Box of 10 | 24 | 0.16 (0.15) | E (F) | 1344 |
| Swinnex 13mm gasket silcon | Pack | 2 | 0.08 (0.07) | E (F) | 112 |
| Printing *Loa loa* photos | Set of prints | 1 | 0.23 (0.21) | E (F) | 56 |
| *Sub-total* |  |  | *1,206 (776)Error: Reference source not found* | *E (F)* |  |
| TOTAL CAPITAL COST | |  | 7,011 (6,199) | E (F) |  |

Part B: RECURRENT COSTS

| INPUT | UNIT | QUANTITY | UNIT COST (USD) | TOTAL COST | COST TYPEError: Reference source not found |
| --- | --- | --- | --- | --- | --- |
| Travel |  |  |  |  |  |
| International flights, visa & registration | Trip | 11 | varied | 8,140 (6,690) | E (F) |
| National flights | Flight | 13 | 400 | 5,200 (5,200) | E (F) |
| Perdiem | Trip | 11 | varied | 2,133 (2,133) | E (F) |
| Car hire | Days | 6 | varied | 769 (769) | E (F) |
| Fuel | Litres | unknown | varied | 185 (185) | E (F) |
| Insurance | Days | 358 | 5.7 | 2,056 | E |
| Accommodation | Days | 6 | 34.3 | 205 (205) | E (F) |
| *Sub-total* |  |  | *18,688 (15,183)Error: Reference source not found* | | *E (F)* |
| Vehicle fuel & maintenance | |  |  |  |  |
| Fuel | Days | 147.6 | 56.8 | 8,383 (8,383) | E (F) |
| Fuel transport to central depot + loading | Delivery | varied | varied | 780 (780) | E (F) |
| Vehicle insurance | Days | 146 | 8.7 | 1,273 (1,273) | E (F) |
| Vehicle maintenance | Vehicles | 3 | varied | 198 (198) | E (F) |
| *Sub-total* |  |  | *10,635 (10,635)Error: Reference source not found* | | *E (F)* |
| Accommodation & sustenance | |  |  |  |  |
| Lodging | Various | n/a | varied | 7,706 (5,206) | E (F) |
| Guarding & cleaning | Various | n/a | varied | 1,464 (1,464) | E (F) |
| Sustenance | Various | n/a | varied | 7,641 (7,641) | E (F) |
| Tent | Tent | 17 | varied | 1,003 (1,003) | E (F) |
| Sleeping bags | Sleeping bag | 17 | 26.8 | 455 (455) | E (F) |
| Sheets | Sheet | 17 | 14.1 | 239 (239) | E (F) |
| Mattresses | Mattress | 17 | varied | 365 (365) | E (F) |
| Towels | Towel | 20 | 7.3 | 145 (145) | E (F) |
| Hygiene & domestic supplies | Various | n/a | varied | 1,669 (1,669) | E (F) |
| *Sub-total* |  |  |  | *20,688 (18,188)Error: Reference source not found* | *E (F)* |
| Survey supplies |  |  |  |  |  |
| Stationary | Various | 1 | 90 | 90 (90) | E (F) |
| Folders | Folder | 10 | 5.7 | 57 (57) | E (F) |
| Printing cartridges | Cartridge | 12 | 46.9 | 563 (563) | E (F) |
| Printer paper | Ream | 4 | 46.9 | 188 (188) | E (F) |
| T-shirts | Shirt | 40 | 6.2 | 246 (246) | E (F) |
| Maps | Map | 6 | 27.5 | 165 (165) | E (F) |
| Latex gloves | Box | 60 | 9.2 | 554 (554) | E (F) |
| Insecticide aerosol | Can | 20 | 2.1 | 42 (42) | E (F) |
| Disinfectant liquid & bleach | Bottles | unknown | varied | 245 (245) | E (F) |
| Tissue | Package | 10 | 1.5 | 15 (15) | E (F) |
| Rubber bands | Pack | unknown | varied | 56 (56) | E (F) |
| Masking tape | Pack of five | 7 | 12 | 80 (80) | E (F) |
| Medium duty sheeting 2m x 50m | Sheet | 3 | 83 | 248 (248) | E (F) |
| Bin liners | Roll | 21 | 4.9 | 107 (107) | E (F) |
| Stamp pad | Pad | 3 | 5.1 | 15 (15) | E (F) |
| Distilled water | 10 L canister | 20 | 29 | 582 (582) | E (F) |
| Reagent bottles | Bottle | 12 | 12.4 | 149 (149) | E (F) |
| Albendazole & praziquantel | Dose | 200 (total) | varied | 13 (13) | E (F) |
|  |  |  |  |  |  |

| INPUT | UNIT | QUANTITY | UNIT COST (USD) | TOTAL COST | COST TYPEError: Reference source not found |
| --- | --- | --- | --- | --- | --- |
| Survey supplies (continued) | |  |  |  |  |
| Slides | Box of 50 | 240 | 5 | 1,200 (1,200) | E (F) |
| Slide labels | Roll | 20 | 13 | 261 (261) | E (F) |
| Glycerol | 1 L bottle | 5 | 199.6 | 998 (998) | E (F) |
| Malachite green oxalate | 25 g bottle | 4 | 80.7 | 323 (323) | E (F) |
| Cellophane sheets | Pack of 50 | 4 | 27.6 | 110 (110) | E (F) |
| Hemastix reagent strips | Box of 50 | 120 | 27.5 | 3,302 (3,302) | E (F) |
| Syringes (10 ml) | Box of 400 | 15 | 51.7 | 775 (775) | E (F) |
| Faecal pots | Box of 400 | 1 | 90 | 90 (90) | E (F) |
| Urine pots | Box of 400 | 1 | 78 | 78 (78) | E (F) |
| Isopore membrane | Box of 100 | 14 | 196.8 | 2,755 (2,755) | E (F) |
| Cover slips | Box of 100 | 12 | 1.5 | 18 (18) | E (F) |
| ICT card tests | Box of 25 | 220 | 103 | 22,678 (22,678) | E (F) |
| Cotton wool | Box of 24 rolls | 1 | 147 | 147 (147) | E (F) |
| Safety lancet | Box of 200 | 23 | 58.5 | 1,346 (1,346) | E (F) |
| Air freight | Shipment | 2 | varied | 5,960 (5,960) | E (F) |
| Customs clearance | Shipment | 2 | varied | 890 (890) | E (F) |
| *Sub-total* |  |  |  | *44,346 (44,346)Error: Reference source not found* | *E (F)* |
| Communication |  |  |  |  |  |
| Mobile & sat phone credit | varied | varied | varied | 1,019 (1,019) | E (F) |
| *Sub-total* |  |  | *1,019 (1,019)Error: Reference source not found* | | *E (F)* |
| Personnel |  |  |  |  |  |
| Management & supervision | Days | 117 | varied | 16,158 (16,158) | E (F) |
| Technical support | Days | 65 | varied | 16,036 (7,276) | E (F) |
| Consultant lab techs | Days | 358 | varied | 18,640 (18,640) | E (F) |
| MoH lab techs | Days | 138 | varied | 6,332 (3,790) | E (F) |
| Drivers | Days | 151 | varied | 2,703 (2,703) | E (F) |
| Guides | Days | 202 | varied | 1,312 (1,312) | E (F) |
| Quality control | Days | varied | varied | 1,048 (205) | E (F) |
| *Sub-total* |  |  |  | *62,230 (50,084)Error: Reference source not found* | *E (F)* |
| TOTAL RECURRENT COST | |  | 157,606 (139,455) | | E (F) |
|  |  |  |  |  |  |
| TOTAL COST (excl. overhead) | |  | 164,617 (145,653) | | E (F) |

1. F = Financial, E = Economic [↑](#footnote-ref-2)
2. This column includes the quantity of items deployed. For example, a total of 143 days of car use was required for the survey, distributed across three vehicles. [↑](#footnote-ref-3)
3. Sub-totals shown are based on actual figures, not rounded ones as shown for each line item. [↑](#footnote-ref-4)
